# Immune System Alterations in Treatment-Resistant Schizophrenia: A Systematic Review of the Current Evidence and Future Directions

**DOI:** 10.3390/ijms27093745

**Published:** 2026-04-23

**Authors:** Marek Krzystanek, Rafał Bieś, Monika Bąk-Sosnowska

**Affiliations:** 1 Department of Clinical and Community Psychiatry and Psychology, Collegium Medicum, WSB University, Cieplaka 1c, 41-300 Dąbrowa Górnicza, Poland; marek.krzystanek@wsb.edu.pl (M.K.); monika.baksosnowska@wsb.edu.pl (M.B.-S.); 2 Department and Clinic of Psychiatric Rehabilitation, Faculty of Medical Sciences, Doctoral School, Medical University of Silesia, Ziołowa 45/47, 40-635 Katowice, Poland

**Keywords:** treatment-resistant schizophrenia, TRS, immune dysregulation, neuroinflammation, cytokines, autoantibodies, biomarkers, immunopsychiatry, clozapine, personalized psychiatry, immunomodulatory therapy

## Abstract

Treatment-resistant schizophrenia (TRS) remains a significant clinical challenge due to limited therapeutic options and a poor understanding of its underlying biology. Recent findings suggest that immune system dysregulation may play a critical role in the pathophysiology of TRS. This systematic review aimed to synthesize current evidence on immunological abnormalities associated with TRS, with a focus on inflammatory markers, immune cell profiles, and the role of autoantibodies, and to explore their potential utility in diagnosis and treatment. A systematic review of the literature was conducted in accordance with PRISMA guidelines, incorporating clinical, molecular, and translational studies on immunological markers in patients with TRS. Included studies assessed cytokine levels, immune cell phenotypes, autoantibodies, genetic factors, and the effects of immunomodulatory therapies. Emphasis was placed on findings differentiating TRS from treatment-responsive schizophrenia. TRS is associated with distinct immune profiles, including elevated IL-6, IL-8, TNF-α, and sCD25 levels, overexpression of CD33 on monocytes and expansion of CD123+ plasmacytoid dendritic cells. Autoantibodies, particularly those targeting glutamatergic receptors, are more prevalent in TRS and decrease with clozapine treatment. Predictive models using IgM autoantibodies and genetic variants show promise for early identification of at-risk individuals. Emerging immunomodulatory treatments such as rituximab, levamisole, and senolytics are under investigation, offering potential for personalized strategies. Immunological dysfunction represents a reproducible and biologically relevant feature of TRS. Integration of immune biomarkers into clinical practice may enhance diagnostic precision and inform personalized therapeutic approaches. Future research should prioritize standardized biomarker protocols and longitudinal studies to validate causal associations and optimize treatment algorithms.

## 1. Introduction

Treatment-resistant schizophrenia (TRS) represents a distinct subtype of schizophrenia characterized by the persistence of symptoms despite the use of appropriate antipsychotic treatment. TRS is defined as the lack of significant clinical improvement following treatment with at least two different antipsychotic medications administered at adequate doses for a minimum duration of six weeks, with confirmed adherence to therapeutic recommendations [1]. Additionally, the current TRRIP (Treatment Response and Resistance in Psychosis) criteria require the presence of symptoms of at least moderate severity, as assessed by standardized rating scales such as PANSS, BPRS, CGI, or FACT-SCZ [2].

In contrast to treatment-responsive schizophrenia, patients with TRS not only exhibit greater resistance to pharmacotherapy but also experience more profound functional impairments. Persistent psychotic symptoms including hallucinations, delusions, and disorganized thinking significantly impair the ability to function socially, professionally, and within family life [3]. Moreover, TRS is associated with more severe cognitive dysfunction, an increased risk of suicidal behavior, comorbid medical conditions [4], lower quality of life, and higher treatment-related costs [5]. Clozapine remains the gold standard in the treatment of TRS, with its efficacy supported by numerous studies and meta-analyses [6,7,8]. However, its use is limited by a range of adverse effects, including potentially life-threatening complications such as agranulocytosis, myocarditis, and pneumonia [9,10]. Importantly, clozapine-induced agranulocytosis is a relatively rare event, occurring in approximately 0.5–1% of patients, while neutropenia is observed in about 3–4%, which should be considered when evaluating its overall risk-benefit profile [10,11]. Current strategies for optimizing treatment emphasize personalized approaches, taking into account genetic factors and individual variations in clozapine metabolism [12].

A clear pathophysiological model of treatment-resistant schizophrenia (TRS) has not been established. Several hypotheses have been proposed to explain the underlying biological mechanisms. One of the most frequently cited is the dopamine supersensitivity hypothesis. According to this concept, chronic blockade of dopamine D2 receptors (DRD2) by antipsychotic medications induces a compensatory increase in receptor density and sensitivity, which may lead to a relapse of symptoms despite continued treatment [13]. This process may result in the need for higher doses, the development of tolerance, and rapid symptom recurrence following drug discontinuation [14]. It is important to note that this hypothesis applies specifically to cases of TRS that emerge after an initially effective treatment response. It does not account for resistance observed from the first episode of schizophrenia, which may involve distinct etiological mechanisms [15]. An alternative to the dopamine supersensitivity hypothesis is the concept of schizophrenia subtypes with varying levels of dopaminergic activity. Research has shown that only a subset of patients with TRS exhibit elevated dopamine synthesis in the striatum. Many individuals demonstrate normal or even reduced dopamine levels, similar to those observed in healthy controls [16]. Notably, such differences may already be present during the first episode of psychosis. This finding suggests that treatment resistance in some patients may stem from distinct and early neurobiological mechanisms [17].

There is also growing evidence suggesting a role of genetic factors in the development of TRS. Associations have been reported between certain polymorphisms in dopamine and serotonin transporter genes, as well as variants in COMT and DRD2 genes, and an increased risk of treatment resistance [18]. Importantly, genomic studies indicate that TRS may involve not only alterations in the dopaminergic system but also other pathways, including the glutamatergic system [19]. Although dopamine dysregulation clearly contributes to the symptoms of schizophrenia in many patients, the failure of antipsychotic dopamine blockade to control symptoms in some individuals suggests that other neurotransmitter systems may also play a significant role in the etiology of TRS. This perspective aligns with the subtype hypothesis. The most prominent among the alternative theories is the glutamate hypothesis. It proposes that dopaminergic overactivity may be a secondary phenomenon resulting from dysregulation of GABAergic and glutamatergic neurotransmission [20].

An increasing body of evidence points to the involvement of inflammatory processes and oxidative stress in the pathogenesis of schizophrenia, including its treatment-resistant form. It has been hypothesized that early-life neuroinflammation, followed by chronic activation of the immune response, may contribute to the development of the disorder [21]. Elevated levels of systemic inflammation may play a particularly important role in the emergence of treatment resistance. Studies have demonstrated distinct inflammatory cytokine profiles in patients with TRS compared to those who respond to treatment, suggesting that such differences may be present from the onset of the illness [22]. Simultaneously, accumulating data indicate elevated oxidative stress in individuals with TRS, measured for instance as increased levels of lipid peroxidation relative to treatment-responsive patients and healthy controls [23]. Neuronal damage resulting from chronic oxidative stress may therefore contribute to the mechanisms underlying treatment resistance. Moreover, patients carrying deletions in genes encoding glutathione S-transferase enzymes, which play a critical role in protection against oxidative stress, may be particularly susceptible to the development of TRS [24]. Although current research in this area remains limited, especially due to the absence of comparison groups in some analyses, the existing findings support the hypothesis that both inflammation and oxidative stress may serve as key contributors to the pathophysiology of TRS.

In this context, increasing attention is being directed toward the role of the immune system, particularly the involvement of autoimmune mechanisms and the potential presence of autoantibodies targeting the central nervous system. The aim of the present study is to provide a systematic review of the literature exploring the possible relationship between these immunological processes and the development of treatment-resistant schizophrenia. Gaining insights into the immune system’s role in the pathophysiology of this subtype of schizophrenia may facilitate the identification of novel diagnostic and predictive biomarkers, enabling earlier recognition of treatment resistance risk. Furthermore, advancing our understanding in this area may pave the way for the development of new targeted therapeutic strategies, offering alternatives for patients who do not respond to standard antipsychotic medications. In the long term, such an approach may contribute to the advancement of more personalized psychiatric medicine in which treatment is tailored to the individual’s biological profile.

## 2. Materials and Methods

The study was conducted in accordance with the PRISMA (Preferred Reporting Items for Systematic Reviews and Meta-Analyses) guidelines [25]. Prior to data collection, it was registered in the PROSPERO database (CRD420251051442).

### 2.1. Inclusion and Exclusion Criteria

The analysis considered original clinical studies, including randomized controlled trials (RCTs), cohort studies, cross-sectional studies, and case reports. Only articles published in peer-reviewed scientific journals and available in full-text format were included. The study population consisted of patients diagnosed with treatment-resistant schizophrenia (TRS), defined in accordance with the Treatment Response and Resistance in Psychosis (TRRIP) criteria [1] or other equivalent classification systems. Eligible studies addressed topics such as the involvement of autoimmune processes in the pathogenesis of TRS, the presence of immunological markers, the effects of immunomodulatory therapies, or the clinical outcomes of immunological interventions. In the case of randomized controlled trials, methodological rigor was required, including appropriate randomization, blinding, and the presence of a control group. Various methods were accepted for evaluating the relationship between autoimmunity and TRS, such as serological testing, neuroimaging, cerebrospinal fluid analysis, and assessment of treatment response. Studies focusing exclusively on other psychotic disorders, such as schizoaffective disorder or organic psychoses, were excluded. Research that failed to clearly distinguish patients with TRS from treatment-responsive individuals was also not included. Review articles, commentaries, conference abstracts, letters to the editor, and publications unavailable in full-text format were excluded from the analysis.

### 2.2. Search Strategy

A systematic literature search was conducted across multiple databases, including PubMed, Embase, Scopus, Web of Science, Cochrane Library, Google Scholar, and ClinicalTrials.gov. The search strategy was based on predefined combinations of keywords and controlled vocabulary terms using Boolean operators. Specifically, the following search string was applied: (“treatment-resistant schizophrenia” OR “schizophrenia”) AND (“autoimmunity” OR “autoantibodies” OR “inflammation” OR “cytokines” OR “neuroinflammation” OR “immunological markers” OR “NMDAR antibodies” OR “GAD65” OR “AMPAR” OR “CASPR2” OR “LGI1” OR “ANA” OR “antineuronal antibodies” OR “B-cell” OR “T-cell” OR “rituximab” OR “cyclophosphamide” OR “corticosteroids” OR “azathioprine” OR “methotrexate” OR “IVIG”).

All retrieved results were merged, and duplicate entries were removed. Articles were screened based on predefined inclusion criteria, and full-text versions of selected studies were reviewed for further analysis.

### 2.3. Eligible Studies

**Figure 1:**
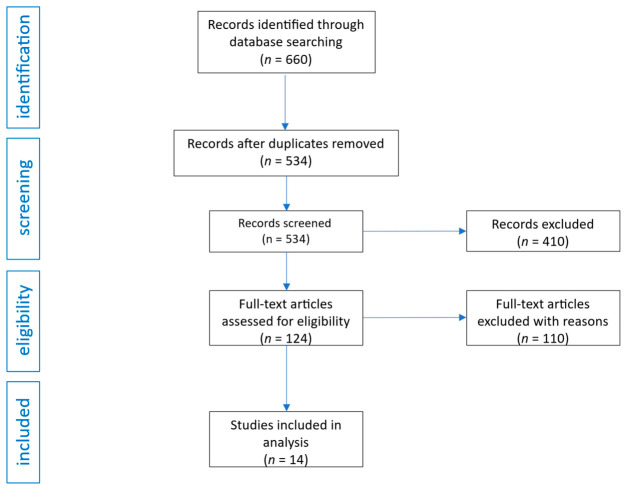
Flow diagram of studies analysis and selection for review.

The database search conducted in accordance with the specified strategy initially identified 660 records. After removing duplicates, 534 unique publications remained. These were screened based on their titles and abstracts, resulting in the exclusion of 410 articles that did not meet the primary inclusion criteria. The remaining 124 studies were assessed in full-text form for their relevance to the review objectives. Following detailed evaluation, 110 articles were excluded based on clearly defined exclusion reasons. In the final analysis, 14 studies met all inclusion criteria and were incorporated into the systematic review. In addition, a search of the ClinicalTrials.gov registry yielded 145 records, of which, 6 were found to meet the inclusion criteria. A visual representation of the selection process is provided in Figure 1.

### 2.4. Data Extraction

From each article included in the analysis, the following data were extracted: first author and year of publication; study design; number of participants; the authors’ conclusion regarding the association between immunological mechanisms and treatment resistance in schizophrenia, which served as the basis for study inclusion in the analysis; and the methodological quality assessment based on the Quality Assessment Tool for Quantitative Studies (QATQS). Particular attention during data extraction was paid to the qualitative interpretation of results and their consistency with the hypothesis of an immunological basis for TRS.

### 2.5. Data Quality

The risk of bias and the quality of quantitative studies were assessed using the Quality Assessment Tool for Quantitative Studies developed by the Effective Public Health Practice Project [26,27]. This method enables a comprehensive evaluation of study designs, including randomized controlled trials, observational studies, and case series, by analyzing eight key methodological domains. These domains include selection bias, control of confounding variables, blinding procedures, data collection methods, and statistical analyses. Each component was rated on a three-point scale: strong, moderate, or weak, based on clearly defined criteria. The overall methodological quality of a study was determined by the number of weak ratings. Studies with no weak ratings were classified as strong, those with one weak rating were classified as moderate, and those with two or more weak ratings were classified as weak. This tool facilitates the identification of both individual limitations, such as lack of randomization, and systemic design flaws, thereby enhancing the transparency and objectivity of quality assessments in the context of evaluating the reliability of results.

## 3. Results

### 3.1. Characteristics of the Included Studies

The analysis incorporated a range of study types retrieved from medical databases, including cohort studies, cross-sectional studies, completed randomized clinical trials, and case reports. The majority of the included publications were published within the past 15 years, although the earliest study dates back to 1987. The total number of participants across all studies was approximately 2000, with detailed demographic and methodological data presented in Table 1. Additionally, clinical studies registered in ClinicalTrials.gov were included in the analysis. These comprised both interventional and randomized trials, with a cumulative sample size exceeding 500 participants. Most of these studies were classified as phase II or phase IV trials, indicating a growing scientific interest in immunomodulatory strategies for the treatment of TRS while also highlighting the need for further early-phase clinical research. Detailed information regarding the included clinical trials is presented in Table 2.

### 3.2. Alterations in Immunological Biomarkers in Treatment-Resistant Schizophrenia

Patients with treatment-resistant schizophrenia exhibit persistent disturbances in inflammatory and autoimmune biomarkers, observed in both cerebrospinal fluid (CSF) and peripheral blood, compared to healthy individuals. Notably elevated CSF interleukin-8 (IL-8) concentrations in TRS patients, relative to controls, indicate intensified neuroinflammatory processes, glial cell activation, and neutrophil chemotaxis within the central nervous system [28]. Regarding cellular phenotypes, significantly increased expression of CD33 on monocytes has been reported in individuals with TRS, potentially contributing to impaired phagocytic function and dysregulated cytokine release [42]. Furthermore, only TRS patients demonstrated elevated levels of CD123+ plasmacytoid dendritic cells [28], which are known to present autoantigens and initiate autoimmune responses.

Hematological analyses revealed consistently increased inflammatory indices namely, neutrophil-to-lymphocyte ratio (NLR) and platelet-to-lymphocyte ratio (PLR) in TRS patients, in contrast to treatment-responsive individuals, whose levels normalized following antipsychotic therapy [29]. Moreover, a trend toward lymphopenia in TRS suggests chronic T cell consumption and activation, features commonly seen in autoimmune diseases [43]. With respect to regulatory cytokines, TRS patients exhibited significantly reduced levels of TGF-β, a key anti-inflammatory cytokine, alongside elevated expression of the transcription factor NF-κB, which activates genes involved in inflammatory and cytotoxic responses [30]. These findings reflect immunoregulatory deficits and a state of heightened immune activation.

Evidence from studies examining the effects of atypical antipsychotics on immunological parameters in TRS patients suggests a dual activation of both pro-inflammatory and anti-inflammatory immune pathways in schizophrenia. Elevated levels of both IL-8 and IL-10 in non-responders to conventional antipsychotics may reflect concurrent monocyte activation and Th2-driven responses [31]. A disrupted blood–CSF barrier, observed in a subset of hospitalized patients, may also play a role in the pathogenic process [32]. Collectively, these data consistently point to pronounced and persistent immune dysregulation in TRS. In particular, diminished numbers and function of regulatory T cells (Tregs), elevated levels of pro-inflammatory cytokines, the presence of antigen-presenting cells and autoantibodies, and sustained increases in peripheral inflammatory markers suggest that a subset of TRS cases may represent an autoimmune or autoinflammatory disease variant [30,31,32].

Further support for this hypothesis is found in the ongoing clinical trial NCT06060886, which aims to identify molecular profiles distinguishing TRS patients from treatment responders. This project utilizes a multi-omics approach encompassing transcriptomics, metabolomics, and cytokine/inflammatory profiling to identify distinctive immunological signatures. Although results have not yet been published, the study design indicates potential identification of biomarkers such as IL-6, TNF-α, and CRP. Another study, NCT05257720, focuses on neurodegeneration-related biomarkers, specifically GFAP and S100B, in TRS patients. Elevated levels of these astrocytic proteins suggest enhanced astrogliosis and neuroinflammation. When considered alongside increased cytokine concentrations, these findings further support the hypothesis that TRS pathophysiology involves overlapping neuroinflammatory and neurodegenerative mechanisms. The extracted data on immunological biomarker alterations have been systematically summarized in Table 3.

### 3.3. Role of Autoantibodies and Autoimmune Diseases in Treatment-Resistant Schizophrenia

Our literature review focused specifically on identifying evidence regarding the potential involvement of autoantibodies in the pathogenesis of treatment-resistant schizophrenia. It has been demonstrated that TRS patients without prior clozapine treatment exhibit elevated levels of autoantibodies targeting glutamatergic receptors, including NMDAR, AMPAR, mGluR3, and mGluR5, whereas patients treated with clozapine show antibody levels comparable to non-resistant groups [33]. This suggests that clozapine may normalize these autoantibody concentrations, potentially explaining its efficacy in TRS through immunomodulatory mechanisms [44]. Complementing this observation, autoantibodies directed against antipsychotic drugs themselves were detected in patients with paranoid schizophrenia exhibiting treatment resistance [34]. The highest antibody titers targeted chlorpromazine, indicating that certain antipsychotics may induce immune responses that could affect their therapeutic effectiveness. This points toward a possible role of drug neutralization, accelerated clearance, or altered metabolism contributing to the development of treatment resistance. Conversely, other studies report inconsistent findings regarding NMDAR autoantibodies in schizophrenia. Some identified their presence in 9.9% of patients, while others found no significant differences compared to controls [45]. Stronger evidence supports the involvement of autoantibodies against cholinergic receptors (m1AChR, nAChR) and serotonergic receptors (5-HT1AR), which are present in a substantial proportion of schizophrenia patients [46,47].

### 3.4. Adjunctive Therapies in Treatment-Resistant Schizophrenia

If the immunological hypothesis of TRS holds true, immunomodulatory agents should influence both disease course and antipsychotic treatment efficacy. Administration of the immunostimulant levamisole to patients with TRS resulted in a significant increase in regulatory T-cell populations, suggesting reversibility of certain immune dysfunctions [37]. Similarly, combined treatment with cycloferon and reamberin markedly improved immunological and clinical parameters in TRS patients [38]. In TRS cases complicated by neutropenia potentially linked to immune responses against clozapine, supportive strategies such as lithium or adenine administration facilitated treatment initiation [39]. Moreover, granulocyte colony-stimulating factor (G-CSF) use in recurrent neutropenia allowed for successful rechallenge with clozapine [40]. These observations highlight immunomodulation as a potentially valuable therapeutic approach in TRS, warranting further well-designed clinical trials [48].

Recent years have seen the emergence of innovative clinical trials testing immunomodulatory therapeutic strategies. One such Phase II study (NCT05240976) investigated the synergistic effect of a drug enhancing NMDA receptor signaling combined with an anti-inflammatory agent in clozapine-resistant TRS patients. This approach aims to alleviate chronic inflammation that may limit the efficacy of both glutamatergic and clozapine therapies. Results expected in 2027 may clarify whether inflammation reduction can modulate clinical response in TRS. Another translational study (NCT03983018) evaluated rituximab, a monoclonal antibody targeting B lymphocytes. This trial not only assessed clinical efficacy, but also secondary endpoints including social functioning, adverse effects, quality of life, cognitive function, and changes in inflammatory biomarkers and B cell subpopulations. It is among the first studies integrating clinical response evaluation with analysis of adaptive immune mechanisms potentially involved in TRS pathogenesis. A third, distinct but equally groundbreaking trial (NCT05838560) examined the efficacy of dasatinib and quercetin combination in psychiatric patients exhibiting features of accelerated aging. Dasatinib and quercetin selectively eliminate senescent cells characterized by a proinflammatory secretory phenotype, which overproduce cytokines such as IL-6, TNF-α, and IL-1β. Given elevated cytokine levels, immunosenescence, and neuroinflammatory changes observed in TRS patients, this therapy may exert anti-inflammatory and rejuvenating effects on the immune system by restoring lymphocyte homeostasis and improving microglial function. Synthesizing the rationale behind these three trials, immunomodulation emerges as a promising novel direction in TRS treatment, paving the way for a more individualized, biologically grounded therapeutic approach.

### 3.5. Immunomodulatory Effects of Clozapine in Treatment-Resistant Schizophrenia

Studies have shown that clozapine increases levels of anti-inflammatory biomarkers such as soluble interleukin-2 receptor (sIL-2R) and CC16 protein, while transiently inducing proinflammatory cytokines including IL-6 and TNF-α [49]. Clozapine also alters immune cell populations, reducing regulatory T cells and plasmablasts. Importantly, clozapine’s efficacy may correlate with baseline inflammatory status—patients with elevated neutrophil-to-lymphocyte ratios (NLR > 1.62) and monocyte-to-lymphocyte ratios (MLR > 0.14) demonstrate better clinical responses [41]. New data from a clinical trial (NCT05741502) conducted at Ohio State University further support the immunological hypothesis for TRS. The study compares inflammatory markers—primarily interleukin-6 (IL-6), as well as hsCRP, IL-1β, IFN-γ, TGF-β, and TNF—between patients receiving clozapine and those treated with other antipsychotics. Preliminary assumptions suggest that clozapine treatment may significantly reduce IL-6 levels, reflecting attenuation of pathological inflammation associated with TRS. Concurrently, psychotic symptoms and suicidal tendencies, previously noted to improve with clozapine, are also evaluated.

Conversely, in a small subset of patients, clozapine may provoke an immune response against the drug itself, manifesting as clozapine-induced agranulocytosis. Although clinically serious, this complication remains relatively rare, affecting approximately 0.5–1% of treated individuals, with neutropenia occurring more frequently but still in a minority of patients [10,11]. Thus, there is a close link between clozapine’s therapeutic effects and its potential immune-mediated adverse reactions. 

## 4. Discussion

The findings of our review support the view that treatment-resistant schizophrenia is characterized by pronounced and persistent disruption of immunological homeostasis. A growing body of clinical evidence points toward the existence of an immunological subtype of schizophrenia, with pathogenic mechanisms extending beyond classical dopaminergic pathways. This perspective is consistent with integrative models of schizophrenia that incorporate genetic, inflammatory, and vascular mechanisms into a unified framework [50]. The distinct pattern of alterations in TRS, such as elevated IL-8 concentrations in cerebrospinal fluid (CSF), overexpression of CD33 on monocytes, expansion of CD123+ plasmacytoid dendritic cells (pDCs), and increased levels of soluble CD25 (sCD25), indicates profound immune system dysfunction. Such abnormalities may lead to insufficient resolution of inflammatory responses, impaired control over autoreactive lymphocytes, and chronic activation of autoimmune processes within the central nervous system [28]. Notably, these inflammatory profiles are not observed in patients who respond well to treatment, which may serve as a foundation for identifying differentiating biomarkers. Moreover, epidemiological evidence indicates a higher prevalence of autoimmune diseases among individuals with schizophrenia, further supporting the role of systemic immune dysregulation [51].

An additional mechanistic aspect that may help interpret the observed immunological alterations in treatment-resistant schizophrenia is the bidirectional interaction between neurotransmitter systems and the immune system. It is well established that peripheral immune cells, including leukocytes, constitutively express receptors and transporters for key neurotransmitters such as dopamine, serotonin, glutamate, and acetylcholine. These receptors are functionally active and participate in the regulation of immune responses, including cytokine production, cell proliferation, and differentiation [52]. Importantly, their expression and signaling properties may be modulated by psychotropic medications, including antipsychotics, antidepressants, and mood stabilizers. Such pharmacologically induced changes may influence immune cell behavior and contribute to downstream effects on inflammatory, endocrine, and neurochemical pathways. In this context, the immunomodulatory effects of antipsychotics—particularly clozapine—may be partially mediated through their action on neurotransmitter receptors expressed on immune cells [53]. This neuroimmune interface provides a plausible biological framework linking neurotransmitter dysregulation with immune abnormalities observed in TRS and may help explain the coexistence of persistent inflammation, altered cytokine profiles, and treatment resistance. Similar mechanisms have been described in previous studies demonstrating the role of neurotransmitter signaling in immune regulation and its modulation by psychotropic drugs [54].

A study utilizing machine learning algorithms to analyze 75 inflammatory markers in plasma demonstrated effective differentiation between patients with TRS, treatment-responsive individuals, and healthy controls [36]. Significant elevations were noted in markers such as IL-6, TNF-α, IL-10, IL-17C, and TNFRSF9, with IL-6 showing particularly high diagnostic value. This approach opens up new avenues for precision diagnostics in psychiatry. A novel and promising research direction is the role of autoantibodies in the pathophysiology of TRS. Their presence—especially those targeting glutamatergic receptors in patients not treated with clozapine—and their reduction following clozapine initiation provide further support for an immune-mediated mechanism [33]. Genetic and epigenetic data related to immune responses support the hypothesis that TRS represents a biologically distinct subtype; these include evidence from DNA methylation studies identifying cellular alterations associated with treatment resistance [35,55]. High sensitivity of predictive models based on IgM autoantibodies and DNA variants indicates the possibility of early identification of individuals at risk for developing TRS [35]. Future studies should focus on transcriptomic and proteomic analyses to identify genes associated with immune hyperactivity in TRS. Insights from other autoimmune conditions suggest that autoantibody production may follow an initial phase of systemic immune activation, which could also be relevant for TRS pathophysiology [56].

Emerging therapeutic strategies aimed at immunomodulation may serve as a basis for developing personalized treatment approaches in TRS. Agents such as levamisole, cycloferon, and granulocyte colony-stimulating factor (G-CSF) show potential in augmenting pharmacological treatment and improving its tolerability [37,38]. Expanding research on targeted therapies, such as rituximab, may lead to more effective modulation of adaptive immune responses and reconfiguration of inflammatory pathways (NCT03983018). Experimental studies using senolytic agents suggest the possibility of restoring immunological balance and reversing features of immunosenescence (NCT05838560). The biomarkers identified thus far may, in the future, serve as predictors of treatment response and as targets for novel therapies, including IL-6 and TNF-α inhibitors. This trajectory marks a promising path toward biomarker-driven, precision psychiatry rooted in biology rather than solely clinical observations. Accordingly, growing evidence highlights the need for routine monitoring of inflammatory markers and consideration of immunological testing in order to optimize treatment strategies and identify patients particularly vulnerable to treatment failure or hematological complications.

Despite the comprehensive scope of the literature reviewed in this paper, several important limitations must be acknowledged. First, the number of studies addressing this issue remains limited, and most of the cited works are characterized by small sample sizes, lack of control groups, or considerable methodological heterogeneity, which hampers direct comparisons of results. Second, most available data are derived from cross-sectional studies, which limits causal inference between immune markers and treatment resistance. Third, few studies directly assess the impact of immune markers on the efficacy of specific antipsychotic agents, limiting their translational utility in clinical practice. Another important limitation is the lack of standardization in biomarker measurement protocols. Laboratory techniques vary between studies, which may contribute to discrepancies in results. Finally, a substantial portion of the data originates from observational studies, increasing the risk of systematic bias due to unaccounted confounding variables. Despite these limitations, this review demonstrates a clear and consistent presence of immune disturbances in patients with TRS, justifying the need for further prospective and interventional studies.

## 5. Conclusions

Based on currently available data, treatment-resistant schizophrenia presents a distinct immunological profile, characterized by chronic inflammation, dysregulated cytokine activity, the presence of autoantibodies, and alterations in immune cell subpopulations. These abnormalities are markedly different from those observed in treatment-responsive patients, supporting the existence of an immunological phenotype of TRS. Advances in the identification of inflammatory and autoimmune biomarkers may enable earlier detection of individuals at risk for TRS, prediction of treatment outcomes, and the implementation of targeted therapies. Immunomodulatory treatments currently under clinical investigation may eventually become the foundation of personalized management strategies for TRS. Further translational studies and integration of molecular data with clinical features are essential for establishing new diagnostic and therapeutic standards based on immunological subtypes of TRS.

## Figures and Tables

**Table 1:** Overview of studies meeting inclusion criteria, focusing on studies on treatment-resistant schizophrenia. The table includes the following information: author and year of publication; study type; number and characteristics of the study population; key findings regarding immunological markers or treatment efficacy; and quality assessment based on the QATQS tool (1—high validity, 2—moderate validity, 3—low validity).

Author, Year	Article Type	Participants	Conclusions	QATQS Score
Scheiber et al., 2022 [28]	Prospective cohort study	39 patients with TRS	Elevated IL-8 in CSF, high plasma ferritin levels, increased CD33 expression on monocytes, and expansion of CD123+ pDCs	1
Labonté et al., 2022 [29]	Retrospective cohort study	156 patients with schizophrenia: treatment-responsive, TRS, ultra-TRS groups	NLR and PLR used as inflammatory markers—persistent inflammation observed in TRS patients	2
Kartalcı et al., 2016 [30]	Cross-sectional observational study	20 TRS patients and 20 healthy controls	Decreased TGF-β and increased NF-κB levels observed in TRS	2
Maes et al., 2002 [31]	Prospective cohort study	17 TRS patients, 14 treatment-responsive patients, 7 healthy controls	TRS characterized by elevated levels of IL-8 and IL-10	2
Bechter et al., 2009 [32]	Cross-sectional observational study	39 TRS patients	Blood-CSF barrier dysfunction and increased neopterin levels	2
He et al., 2024 [33]	Cross-sectional observational study	37 TRS patients, 39 TRS patients never treated with clozapine, 35 responsive patients	TRS associated with elevated levels of glutamate receptor autoantibodies	3
Govorin et al., 1991 [34]	Comparative cohort study	112 patients with paranoid schizophrenia (acute phase, chronic exacerbation, and TRS groups)	Antipsychotic drug antibodies (DATs) were present in all groups, highest in TRS patients; DATs may affect treatment response, particularly with chlorpromazine	1
Stassen et al., 2007 [35]	Original study—molecular genetic analysis	1003 psychiatric patients including TRS patients	Predictive model based on genetic analysis and neural networks suggested that elevated IgM indicates inflammatory TRS processes	1
Yee et al., 2025 [36]	Cross-sectional observational study	49 treatment-responsive, 68 TRS, 29 ultra-TRS patients	TRS subgroups showed elevated inflammatory marker profiles including IL-6, TNF, IL-10, IL-12B, CST5	2
Mikheeva et al., 1987 [37]	Randomized clinical trial	13 TRS patients	Levamisole increased T-suppressor lymphocyte count (from 16.6% to 22.8%); no such change in placebo group	2
Maruta et al., 2011 [38]	Randomized clinical trial	90 TRS patients in treatment group, 70 in treatment-responsive group	Adjunctive cycloferon and reamberin with standard therapy led to clinical improvement, more remissions, reduced toxic syndrome	1
Takanobu et al., 2022 [39]	Case report	1 TRS patient with neutropenia	Successful clozapine initiation in neutropenic patient using supportive strategies including immunomodulatory agents (lithium carbonate, adenine)	3
Béchard et al., 2021 [40]	Case report	8 TRS patients with clozapine-induced neutropenia	Use of filgrastim (G-CSF) enabled re-initiation or continuation of clozapine in some neutropenic patients	3
Llorca-Bofí et al., 2024 [41]	Prospective cohort study	32 TRS patients	Higher baseline NLR (neutrophil/lymphocyte) and MLR (monocyte/lymphocyte) ratios correlated with better clinical response to clozapine after 8 weeks	2

**Table 2:** Overview of registered clinical trials that met the inclusion criteria. The table includes the following columns: ClinicalTrials.gov Identifier; Clinical Trial Phase (I–IV); Study Design; Number of Participants; Intervention; Mechanism of Action—understood as the immunological basis or mode of action of the intervention; Immunological Findings—key conclusions regarding inflammation or immune response.

Study ID	Clinical Trial Phase	Study Design	Participants	Intervention	Mechanism of Action	Immunological Findings
NCT05741502	IV	Open-label, parallel groups (clozapine vs. other antipsychotics)	60	Clozapine ≥ 6 months vs. other antipsychotics	Comparison of IL-6, TNF-α, IFN-γ levels	Clozapine may exert immunomodulatory effects
NCT05240976	II	Randomized, triple-blind	80	NMDA enhancer + anti-inflammatory vs. placebo	Testing synergy between glutamatergic and immune pathways	Inflammation may limit TRS treatment efficacy; immunomodulation may enhance it
NCT05838560	II	Randomization, details not specified	Ongoing recruitment	Dasatinib + quercetin (senolytics)	Elimination of SASP cells; anti-inflammatory and anti-aging effects	Reduction of chronic inflammation via removal of senescent cells (IL-6, IL-1β, TNF-α)
NCT06060886	IV	Randomized, open-label, multicenter	244	Aripiprazole vs. Paliperidone	Systemic biological analysis	Cytokine and metabolite profiling may identify immune signatures in TRS patients
NCT05257720	Observative	Prospective, 3 groups (TRS, remission, control)	180	No intervention—biomarker analysis	Measurement of GFAP and S100B	Elevated GFAP/S100B may indicate involvement of inflammation and neurodegeneration in TRS
NCT03983018	I	Randomization details not specified	9	Single rituximab infusion (1000 mg)	Evaluation of inflammatory markers	Potential correlation between clinical response and inflammation reduction (B cells, cytokines)

**Table 3:** Selected immunological biomarkers in treatment-resistant schizophrenia. TRS—Treatment-resistant schizophrenia; CSF—Cerebrospinal fluid; Treg—Regulatory T cells; NLR/PLR—Neutrophil-to-lymphocyte ratio/Platelet-to-lymphocyte ratio (markers of systemic inflammation); NF-κB—Nuclear factor kappa B, a transcription factor involved in inflammatory gene expression.

Biomarker	Change in TRS	Comparison	Immunological Significance	Reference
IL-8	Significantly elevated	Higher than in healthy controls	Neuroinflammation, neutrophil chemotaxis, glial activation	[28]
CD33+ on monocytes	Highly expressed	Higher than in healthy controls	Phagocytosis inhibition, dysregulated cytokine regulation	[28]
CD123+ on plasmacytoid dendritic cells	Increased count	Higher than in healthy controls	Autoantigen presentation, role in autoimmunity	[28]
sCD25	Significantly elevated	Higher than in healthy controls	Treg cell dysfunction, impaired immune tolerance	[28]
Ferritin	Elevated	Higher than in healthy controls	M1 macrophage activation, oxidative stress	[28]
NLR/PLR	Elevated	Normalized only in treatment-responsive patients	Persistent inflammation despite treatment	[29]
TGF-β	Significantly decreased	Lower than in healthy controls	Impaired immunosuppression, Th1/Th2 imbalance	[30]
NF-κB	Elevated	Higher than in healthy controls	Chronic inflammatory activation, immune response dysregulation	[30]

## Data Availability

No new data were created or analyzed in this study. Data sharing is not applicable to this article. The PRISMA check list is available in the Supplementary Materials.

## Supplementary Materials

The following supporting information can be downloaded at: https://www.mdpi.com/article/10.3390/ijms27093745/s1. Reference [57] is cited in the supplementary materials.
